# Experiencing the community of inquiry framework using asynchronous online role-playing in computer-aided instruction class

**DOI:** 10.1007/s10639-021-10670-5

**Published:** 2021-08-14

**Authors:** Kasiyah Junus, Harry Budi Santoso, Mubarik Ahmad

**Affiliations:** Faculty of Computer Science, Universitas Indonesia, Kampus Baru UI Depok, Depok, Jawa Barat, 16424 Indonesia

**Keywords:** Asynchronous, Collaborative learning, Community of inquiry, Discussion forum, Role-playing

## Abstract

This current study investigates the use of online role-playing, in an online discussion forum, in learning the community of inquiry framework – an area of learning covered in the Computer-Aided Instruction (CAI) course, an elective course for Computer Science undergraduate students at Universitas Indonesia. The participants were divided into different roles. Each group was triggered to discuss the implementation of online collaborative learning. A mixed-methods approach was utilised to analyse the qualitative and quantitative data. The result of content analysis exhibited students implementing all the components of the CoI framework. Teaching presence was the rarest, as students were focused on delivering their ideas. Social presence appeared in almost all messages since it is the easiest, and students can feel the impact immediately. The discussion moved to the integration phase but did not proceed to resolution. This study suggested some recommendations and future research topics.

## Introduction

Experts agree that online collaborative learning has the potentials to develop students' critical thinking skills. For a discussion to be meaningful and sustained, each individual needs to play an active role as a member of a community of inquiry. The community of inquiry framework (CoI) is a framework designed to guide meaningful online discussions; it explains the online collaborative learning process within a community of inquiry as to the dynamics of social presence (SP), cognitive presence (CP), and teaching presence (TP) (Garrison et al., 1999).

Sustained and meaningful online collaborative learning requires students to play their part in a knowledge construction discourse. Although they already have the technical readiness for learning in an online learning environment, students may not have the necessary skills for interacting meaningfully within their learning community (Junus et al., 2019). Moreover, these skills cannot be acquired trivially. By applying the CoI framework, the necessary skills can be taught to students to fulfill their roles in online collaborative learning. Although the CoI framework has been implemented in several universities in Indonesia (Pratiwi et al., 2016; Yandra et al., 2021), studies on instructing students in the CoI framework to improve their preparedness to collaborate online in Indonesia are still limited. This current study used the CoI framework for two essential purposes: the study's theoretical framework and the teaching of the framework in the online role-playing context.

The CoI is one of the areas of learning covered in the Computer-aided Instruction (CAI) course, an elective course offered by the Faculty of Computer Science Universitas Indonesia. The objective of this learning area is to prepare and equip students to play a more active role in an online discussion through SP, CP, and TP. The learning strategy chosen to deliver the CoI framework is that of role-playing, which will be followed by discussion and reflection. This strategy was selected as the learning objectives involve changing behaviours and forming interpersonal skills in an online collaborative learning environment. The following discussion and reflection are designed to allow students to articulate and internalise their learning experience using the CoI framework.

### Problem statement

The CoI framework guides learners and instructors in promoting a meaningful and sustained online discussion. Education experts agree that role-playing activity has the potential to develop interpersonal skills in an online collaborative learning environment. Ideally, online role-playing on the CoI framework would be able to improve students’ participation in sustained discussion through social, teaching, and cognitive presences. Encouraging student participation in discussion forums is a challenge in online collaborative learning. Participation rates are low for various reasons, and students do not yet understand their role as group members in online collaborative learning.

The CoI framework is used to guide students in carrying out their roles to participate in text-based interactions. The CoI framework is a new concept for students. They may not have realized the importance of presenting the three components of the CoI framework. Participating in online role-playing maybe also the first experience for students, so they need to adapt to this method. In this study, online role-playing was applied to prepare, implement, and strengthen the experience through discussion and reflection. The selection of roles to be played is adjusted to the actual situation setting in an online learning environment of a faculty that will implement online collaborative learning. It is expected that students can build meaningful discussions guided by the CoI framework and gain meaningful learning experiences.

### Research questions

This study investigates how students perform role-playing and project themselves in social, teaching, and cognitive presences and how students interpret their learning experiences. The following research questions guide this study:

- RQ1: What characteristics of role-playing did the students exhibit?
- RQ2: What are the patterns of social presence, cognitive presence, and teaching presence for each group?
- RQ3: How are the students’ perceptions of the role-playing learning experience and their learning satisfaction?

## Relevant literature review

### Role-playing

Role-playing is an instructional technique, taking on the role of a character, the assumption of a part, or the representation of a type in a pretend situation (Collins & O’Brien, 2003). This instructional strategy is intended to stimulate critical thinking as students learn new skills. Role-playing is a teaching strategy that fits within the social family of models (Joyce et al., 2000). These strategies emphasise the social nature of learning and consider cooperative behaviour as stimulating students, both socially and intellectually (Jarvis et al., 2002). They also emphasise that the role-playing teaching strategy is advantageous for both students and instructors: raising student interest in the topic, increasing involvement in the learning process, and teaching empathy and understanding for different perspectives.

Greenberg and Eskew (1993) presented a review article on how role-playing has been used to learn about behaviour in organisations. They categorised three critical dimensions by which previous papers may be characterised: Level of Involvement (passive-active dimension), Role Being Played (self-other), and Degree of Response Specificity (free and spontaneous manner – highly restricted, specified manner).

Online role-playing has the potential to be an effective student-centered learning activity that can contribute to a successful and highly enjoyable learning experience (Bender, 2005; Erturk, 2015). The online role-playing strategy provides students with the opportunity to interact meaningfully with the learning material and each other. Instructors are benefited from gaining more excellent knowledge and understanding of each student (Bender, 2005). Hence, the instructors are better equipped to help students achieve the learning objective.

Erturk (2015) proposed three specific teaching approaches that can be incorporated into a lesson plan to implement role-playing: catering for learner needs, active learning approaches (learning by doing), and feedback to learners. Learners gain feedback from their instructors and provide feedback to one another – prompt and meaningful feedback is crucial to improving learning.

Role-playing requires careful preparation, monitoring, and evaluation to achieve the learning goals. Cherif et al. (1998) suggested four stages of role-playing, each of which can also be applied to online role-playing: preparation for the activity by the instructor; classroom preparation for the students; the role-playing; and after the enactment.

### The CoI framework

Online collaborative learning is an active learning method in which students solve problems collaboratively in an online learning environment; students can define and formulate problems independently and brainstorm, negotiate, answer questions, explain and debate to construct knowledge. Garrison and Vaughan (2008) emphasised the importance of a theoretical framework in adopting a particular technique, such as online collaborative learning, to prevent distortion due to the gap between theory and practice.

The CoI is a coherent framework that provides both a means to shape practice and reflect upon or evaluate outcomes (Garrison, 2011; Garrison & Vaughan, 2008). Garrison and Arbaugh (2007) stated that the CoI framework has proved helpful in guiding research and practice in online higher education. Castellanos-Reyes (2020) highlighted that the CoI framework is one of the most extensively used frameworks in online teaching and learning by researchers across the globe.

The ability to build positive learning environments is reflected in SP. Garrison et al. (1999) categorise SP into three subcomponents: emotional (affective) expression, such as personal expressions and values; open communication; and group cohesion. TP also consists of three subcomponents: instructional design and organisation, facilitating discourse, and direct instruction. The heart of discourse, CP, is operational critical thinking comprised of a triggering event (problem identification for further inquiry), exploration, integration (synthesising and making meaning from ideas formed during exploration), and a resolution (defend the solution or apply the new skills and knowledge learned into a different context) (Garrison et al., 1999).

Alavi and Taghizadeh (2013) detailed five reasons the CoI framework was selected in their technology-assisted collaborative learning research: The CoI framework explains the process of deep and meaningful collaborative learning experiences; it guides researchers to conceptualise complex interactions between participants in online learning; it outlines the behaviours and processes needed in knowledge construction in the asynchronous learning environment; it models online collaborative learning processes; it explains online learning experiences that continue to be developed and studied. Fiock (2020) stated that the CoI framework is one of the most widely used frameworks for building communities online.

Junus et al. (2019) applied cognitive apprenticeship to teaching students the CoI framework, integrated within a Linear Algebra course. The study revealed that students equipped with the CoI framework experienced increased levels of communication skills, self-regulation, co-regulation, and learning strategies. Research on how to teach the CoI framework as an area of learning within different subject matters is currently limited. In response to this research gap, this current study examines the application of role-playing as a method to teach the CoI framework. The CoI framework is one of the CAI course areas, intending to equip students with the necessary skills to interact in online collaborative learning by implementing the CoI framework.

Research on equipping the CoI framework to improve learning readiness and critical thinking skills have been done before, such as in Boris and Hall (2005) and Santoso (2014). The methods used in these studies were relatively similar, explaining to students about the framework at the beginning of a course. Students were expected to apply their knowledge to solve problems in an online collaborative learning setting. The results showed an improvement in participants' critical thinking (Boris & Hall, 2005). On the other hand, however, the study conducted by Santoso (2014) showed that dissemination of the CoI framework was not proven to improve students' critical thinking skills significantly.

Forsythe (2020) created the community of inquiry based on the CoI Model as a course topic. One of the objectives is that the participants should interact with other class members to build their learning community. The course looks at the community of inquiry and the CoI framework from the perspectives of learners and teachers. It is expected that the knowledge and skills in future practices. The course activities take place in an online discussion forum combined with reflection.

## Context of the study

### CAI class description

The CAI course is an elective course offered to third-year Computer Science students at Universitas Indonesia. This three-credit course covers the following topics: a historical review of learning and technology; learning: from speculation to science; instructional fundamentals; pedagogy concepts and online pedagogy; objectivism learning theory and behaviourism; cognitivism; constructivism; metacognition and self-regulated learning; critical thinking; online collaborative learning theory; the CoI framework; multimedia learning; course design for online learning; authoring tools and learning management systems; contemporary issues.

The CAI class was delivered in a blended learning model. The online interaction was facilitated through a learning management system called the Student-Centered e-Learning Environment (Hasibuan & Santoso, 2005). Asynchronous online discussion is incorporated as part of class instruction, designed to actively engage students in interactions with their lecturers and fellow students. The evaluation consists of weekly reflections, mid-term tests, and a final group project. In the final project, groups of five students were asked to propose a design for online training, including the online course design and prototype development; they were expected to apply the theories they had learned, including the CoI framework.

Based on the literature review, role-playing has been recognized as one of the active learning techniques. This section elaborates on the advantages of role-playing and its procedures in an online learning class context. The advantages of applying role-playing in CAI class are the following.

1. Role-playing is relevant to the topic and learning objectives, and the topic can be packaged as a problem to be discussed using the role-playing method. Students can immediately apply new knowledge of the CoI framework, which also serves as a guide for meaningful interaction in an online collaborative learning environment. Students practice their skills in meaningful discussion behaviours, under the CoI framework, in real situations; thus, making it easier for them to apply knowledge about the CoI framework in collaborative learning.
2. Role-playing in online discussion forums provides equal opportunities for all students to participate actively – it provides opportunities for interactions between individuals in the group.
3. Role-playing is a new experience for students – unusual learning activities ignite enthusiasm. Students are able to experience the problem from the perspective of those in the community they are playing in, which differs from their daily roles (except for the student group). In partaking in these various roles, students become more enthusiastic in carrying out their roles, so student involvement is high.
4. Students are provided the opportunity to express their feelings and learning experiences to the faculty, particularly those that apply online learning. For example, students have experienced difficulties related to the learning changes caused by the COVID-19 Pandemic; they are able to present these experience under their role (such as a parent relaying their child's experience or a member of the graduate user team communicating the difficulties they experienced as a student), rather than as the student they are. Students internalise the experience from the perspective of the group context being portrayed.
5. Role-playing in an asynchronous online discussion environment accommodates introverted students and students hampered by face-to-face discussions that demand spontaneity and quick-thinking skills. Online discussions that utilise the role-playing method provide students with the opportunity to think and prepare for their contributions in advance.
6. Role-playing facilitates students in expressing their thoughts and feelings freely. The group discussion's atmosphere of discussion within the community context is not the same situation as that of a group discussion during classroom learning. In the role-playing context, participants create a discussion environment situation and adjust; this allows students to build a comfortable and trusting environment where they contribute towards attaining their common goals. Ahmadaliev et al. (2018) emphasised the importance of such activities driven by the online community.
7. Role-playing is followed by a discussion analysing the learning process and achievements. Thus, students are trained to observe and analyse situations in the learning process they and others have navigated.

### Online role-playing and its procedures

The following procedure was applied to conduct role-playing as our learning technique. The procedure consisted of several steps: Preparation, Orientation, Implementation, and Internalisation. The steps in conducting online role-playing are shown in Table 1. The orientation before the role-playing included two aspects: a brief introduction to the CoI framework to guide the role-playing (online discussion) process and the role-playing process. A familiar problem triggered the role-playing, namely implementing collaborative online learning in an institution where they are pretended to be the stakeholders. Students did not discuss the CoI framework, but they were encouraged to remind other members to apply the components of the CoI framework as a part of TP. An in-depth discussion about the CoI framework was conducted after the role-playing.

1. Preparation for the activity by the instructor
  Students are evenly divided into six groups. Each group is given one of the following six roles: students, lecturers, academic secretariat and information technology (IT) division, parents of the students, association of graduate users in the field of IT, and, lastly, faculty management.
2. Task orientation
  Substance orientation begins with an introduction using online discussion forums:
  “*The topic we cover is Community of Inquiry framework. For the discussion to be meaningful and sustainable and support learning outcomes, participants need to adopt and apply a social presence, cognitive presence, and teaching presence. To understand more deeply, you are required to read the papers that have been uploaded on the online class page. The learning strategy that we use is role-playing in the forms of online discussion. Each group has a role to play in an online discussion forum. A discussion trigger will be given to each group. During the discussion, the students are expected to apply the CoI framework to guide the discussion. Enjoy the process!*”
  The substance orientation was followed by a brief explanation of the CoI framework and provided a reading assignment consisting of articles on the CoI framework. The orientation of the role-playing process was performed by explaining the formation of the groups, the roles of the individuals within a group, and the task of each group. In online role-playing, the explanation (orientation) must be explicit. More importantly, teaching the CoI framework requires modeling by lecturers. The lecturers demonstrate examples of implementing cognitive, teaching, and social presences in their online interactions with the students.
3. The role-playing
  The following role-playing task initiates each focus group discussion:
  * “Our faculty plans to implement online collaborative learning (OCL) in every class next semester. As you know, so far, only a few classes have implemented OCL. This learning strategy is relatively new for some students and lecturers. The University Chancellor asks your group to give recommendations regarding the plan. “*
  The reasons for choosing the issue of online collaborative learning are as follows: it is a relatively new strategy for some lecturers and students at universities, students have individual and existing experiences of online discussion, it can be viewed from a range of perspectives, and it can be designed to resemble real-life situations of students closely. Moreover, the CoI is a framework designed to shape the community in an online learning environment.
4. Internalisation
  This phase is achieved through discussions that provide students with the opportunity to articulate their experiences. Lecturers support this by providing feedback and facilitating discussion, and students also receive feedback from their peers. The steps in conducting online role-playing are shown in Table 1.

**Table 1:** Steps in conducting online role-playing

Preparation	Orientation	Implementation	Internalisation
Lecturers’ activities			
• Determination of role-playing scenarios and rules of the game • Uploading reading materials for independent study • Group formation and role assignment	• Content orientation: explaining briefly about the CoI framework • Process orientation: briefing about role-playing process in a discussion forum, learning strategies and expectations	• Triggering each group based on individual roles • Monitoring the process and facilitating discourse with minimum intervention • Fading away as students become more engaged	• Initiating class discussion after completion of the role-playing to articulate the learning experience. Facilitating each group to carry out self-evaluation • Providing feedback
Students’ activities			
• Joining a group and getting to know other group members	• Attending the orientation • Reading the material about the CoI framework	• Playing the role in asynchronous online discussion forums	• Offering feedback to other students • Engaging in group self-evaluation • Composing individual reflection to deepen understanding and organise newly learned knowledge

In terms of the degree of response specificity, participants were free to express their ideas; they were expected to participate freely and spontaneously. Based on the role being played, those playing the role of students played their own real-life role (self-role) while the group's remaining members played other roles (Table 2). It is generally easier to play a real-life role than someone else's. The more familiar students are with the task set, the easier they find it to play.

**Table 2:** Roles played

Group	Person Played	
	Self	Other
Students	√	
Lecturers		√
Academic Secretariat and IT division		√
Parents of The Students		√
Association of Graduate Users in the field of IT		√
Faculty Management		√

## The method

A mixed-methods approach was utilised to analyse the qualitative and quantitative data (Buchholtz, 2019; Nieswandt & McEneaney, 2009; Ponce & Pagán-Maldonado, 2015). A coding template that describes indicators of the components of the CoI framework was used to analyse the discussion transcripts.

### Participants

Participants in this study were CAI students offered by the Faculty of Computer Science at Universitas Indonesia. Before the learning, students were free to decide whether to participate in the study. No consequences or benefits were applied to the choices regarding participation. The enactment in role-playing was voluntary and occurred naturally, without instructor direction. The discussion element was conducted in a free and spontaneous manner. During the role-playing, instructor intervention was limited.

### Data collection

There are two types of data collected for this study: qualitative data and quantitative data. Qualitative data are obtained from discussion transcripts, reflections, and questionnaires (open-ended questions) of the students. We extracted the transcripts in role-playing from online discussion forums in the 2^nd^ semester of 2019/2020. We used thematic content analysis to measure elements of CoI and the characteristics of the role-playing. At the end of the semester, we asked the students to reflect on their learning experience in the role-playing session. Also, we asked them to give suggestions, criticisms, ideas, and comments. These qualitative data are used to measure the students’ opinions about the role-playing implementation.

We also used quantitative data in this study. We extracted the data from online discussion forums in the CAI classes (2^nd^ semester 2018/2019, 1^st^ semester 2019/2020, and 2^nd^ semester 2019/2020). The data were grouped based on roles: for each group, the number of messages and the average number of sentences per posting were counted. These numbers indicate the level of involvement (passive-active dimension). Moreover, we also collected the questionnaires from students at the end of the 2^nd^ semester 2019/2020 to measure their students’ learning experience and satisfaction. The description of data used in this study is shown in Table 3 below.

**Table 3:** Data description

Type of data	Sources	Semester	Analysis	Measurement
Qualitative	Discussion Transcript	2^nd^ of 2019/2020	Thematic content analysis	Cognitive presence, teaching presence, and social presence
		2^nd^ of 2019/2020	Thematic content analysis	Characteristics of the role-playing
	Reflection	2^nd^ of 2019/2020	Thematic content analysis	Obstacles and students’ opinion
	Lecturer evaluation by students (open-ended questions)	2^nd^ of 2019/2020	Thematic content analysis	Suggestions, criticisms, ideas, and comments
Quantitative	Discussion forums	2^nd^ of 2018/2019, 1^st^ of 2019/2020, and 2^nd^ of 2019/2020	Number of postings and average sentences per message	Students’ involvement
	Lecturer evaluation by students (6-Likert Scale Questionnaires)	2^nd^ of 2019/2020	Descriptive statistics	Students’ learning experience and satisfaction

### Data analysis

Information extracted from the data collected over the three consecutive semesters is as follows: the level and pattern of involvement in each group, the number of students posting, the average number of sentences for each message in each group, and the average number of sentences each group. This information is intended to describe the level of activity for each group across each semester. The qualitative data (discussion transcripts) was taken from the CAI class of the Second Semester 2019/2020. The data were analysed using the quantitative content analysis method (Rourke & Anderson, 2004), and categorisation, based on themes, was used to investigate the pattern of the presence of the components of the CoI framework.

The coding template that describes the indicators of the components of the CoI, developed by Garrison et al. (1999), was utilised. This data analysis aimed to answer RQ1 (*What characteristics of role-playing did the students exhibit?*) and RQ2 (*What are the patterns of social presence, cognitive presence, and teaching presence for each group?*). The quantitative content analysis adhered to the following steps: sampling, determination of the unit analysis, discussion of coding procedure between two coders and coding protocol exercised, coders working independently, comparison of coding results, coding decision, and analysis.

Three hundred and six (306) messages were taken as the sample. The unit analysis in this study is a message. The sentence, paragraph, and theme are not suitable as the unit analysis, as some messages are long, and they are not presented in paragraph format. The samples were coded by two coders – lecturers for the CAI course – that have implemented the CoI framework for the past three years (they are also educational researchers and authors).

Messages were coded based on the existence of the SP, TP, and CP. Following this, the CPs were further coded into their sub-components: triggering event, exploration, integration, and resolution. Before coding independently, the coders determined the coding procedure and exercised the coding protocol. For each unit analysis, the coders sought the indicator of the CoI components: SP, TP, and CP. If at least one indicator appeared for each CoI component, they labelled the message with a ‘1’; otherwise, they labelled it ‘0’. One message may contain SP, TP, and CP simultaneously. The coding results of the two coders were compared and calculated using Fleish's Kappa to determine interrater reliability. If disagreement occurred, the coding decision was discussed and agreed upon by both coders.

The thematic content analysis was intended to identify the following indicators: (1) how individuals played their role, for example, how they identified themselves as a member of their group (2) whether they conveyed ideas, obstacles in the OCL or strengths in the OCL, according to the perspective of the role they played; (3) whether they related to the situation encountered during the discussion, such as the COVID-19 pandemic; (4) whether they explicitly mentioned the CoI framework or its components; and (5) how students identified themselves based on the characters being played.

In addition, to answer the RQ 3 (*How are the students’ perceptions of the role-playing learning experience and their learning satisfaction?*), collected data from the reflection of learning and lecturer evaluation questionnaires were analyzed using descriptive statistics and content analysis.

## Findings

The findings are presented to answer three research questions: (RQ1) What characteristics of role-playing did the students exhibit?; and (RQ2) What are the patterns of social presence, cognitive presence, and teaching presence for each group? (RQ3) How students’ perceptions of the role-playing learning experience and how their learning satisfaction?

### Students involvement in role-playing

Before answering the research questions, we used quantitative analysis to investigate the involvement of students in asynchronous role-playing based on their online discussion transcripts. The involvement of the students in online discussion shows in Table 4. Students of the second semester 2018/2019 class had the smallest average number of posts (2.2). The other classes had similar averages of 5.87 and 5.67. The average number of posts for a group varied, as shown by the last column of Table 4.

**Table 4:** The number of students’ postings in role-playing discussions

Group	Number of Postings			Average
	2^nd^ semester 2018/2019	1^st^ semester 2019/2020	2^nd^ semester 2019/2020	
Students	25	64	46	45
Lecturers	24	57	67	49.3
Academic Secretariat and IT Division	19	50	38	35.7
Parents of the Students	23	56	42	40.3
Association of Graduates Users in the Field of IT	19	68	56	47.7
Faculty Management	27	57	57	47
The average numbers of posts	22.8	58.7	51	44.2
The average numbers posts per person	2.2	5.87	5.67	4.58

Note. The discussion duration in 2^nd^ semester 2018/2019 and 1^st^ semester 2019/2020 are seven days with ten group members. Meanwhile, in 2^nd^ semester 2019/2020 is five days with nine group members

The duration of discussions ranges from five to seven days. The numbers of group members are between nine and ten students. The class of the first semester 2019/2020 had the highest average number of posts and the average number of posts per student. The comparison of the number of postings is shown in Table 4. The difference in the discussion duration and the number of group members did not correlate with the average number of posts.

The academic secretariat and IT Division has the lowest frequency of posts (the least active). Its role is non-academic and indirectly relates to the implementation of OCL. Students found identifying the tasks of the supporting staff roles difficult and providing recommendations to faculty leaders. Moreover, this group was the last group formed. Students who had not previously been placed in any of the other groups joined this one. There was no dominant member in this group.

Next, the number of sentences in a message was investigated. The number of sentences in a message generally indicated the number of ideas conveyed. Moreover, they could also reflect the fluency with which students conveyed their ideas. Table 5 below presents the average message length for each group.

**Table 5:** The average number of sentences per message in role-playing discussions

Group	Average Number of Sentences per Message			Average
	2^nd^ semester 2018/2019	1^st^ semester 2019/2020	2^nd^ semester 2019/2020	
Students	6.84	7.29	6.63	6.92
Lecturers	6.92	7.61	4.92	6.48
Academic Secretariat and IT Division	6.89	7.34	5.74	6.66
Parents of the Students	5.30	8.32	6.98	6.87
Association of Graduates Users in the Field of IT	7.16	8.54	5.52	7.07
Faculty Management	6.40	9.56	5.86	7.27
The Average of Each Semesters	6.58	8.11	5.94	6.88

Note. The discussion duration in 2^nd^ semester 2018/2019 and 1^st^ semester 2019/2020 are seven days with ten group members. Meanwhile, in 2^nd^ semester 2019/2020 is five days with nine group members

It is generally easier to play one’s own role than acting in the role of the others; however, the data showed that the student group was neither the most active nor did it have the highest average message length. The existence of dominant members in a group may contribute to the level of activity of the group. The faculty management group of the First Semester 2018/2019 had two dominant members – the dominant members are the most active and the most frequently referred to or mentioned in discussions. The other groups had, at most, one dominant member. This description goes some way to answering the research question regarding the level of student involvement in role-playing activities.

### The characteristics of the role-playing

Answering the research question RQ1: “What characteristics of *role-playing* did the students exhibit?” we used thematic content analysis based on the discussion transcripts. The discussion transcript was investigated in greater depth, based on the following themes: (1) how individuals played their role and whether they identified themselves as a member of the group; (2) whether they conveyed ideas from the perspective of their role, conveyed obstacles in OCL and conveyed OCL strengths; (3) whether they related to the situations encountered during the discussion – the COVID-19 pandemic; and (4) whether they explicitly mentioned the CoI framework or its components. The results are presented in Table 6.

**Table 6:** Thematic content of the discussion transcript

Themes/ indicators	Group	Exemplary messages
Playing the role accordingly: • identifies themselves as a member of the group • mentions other members using specific title • presents points of view according to the role	All groups	*Good afternoon, Dean* *Based on the Dean's statement regarding Online Collaborative Learning (OCL), are there any other advantages in implementing collaborative learning compared to regular online teaching during this distance learning session?* *Thank you, Academic Manager* *[Faculty management]*
Connecting the discussion to the current issue: the COVID-19 pandemic	Lecturers Academic secretariat and IT Division Association of Graduates Users in the field of IT Faculty management	*Good afternoon,* *Dear Professors, I agree with Ms. Lulu's opinion, that the COVID-19 pandemic is an extraordinary event that requires faculty to take action regarding the implementation of teaching and learning activities* *[Lecturers]*
Conveyed obstacles and strengths of the OCL process	Students Lecturers Academic secretariat and IT Division Parents of students Faculty management	*Good afternoon Mr XX* *I think the obstacles my children Shelly and X, Mr XX’s son, are quite similar, the lack of interaction in discussion. From the system's view, I think OCL is good for students, because OCL requires discussion among participants, increasing interaction while practicing our kids’ critical thinking skills. In my opinion, the role of the faculties should be able to be a facilitator for OCL activities, and parents can be a partner for kids’ discussion. At least, just being a listener, like my daughter to me (haha: D) is enough to help stimulate her confidence* *Regards, (Shelly's father)[Parents of students]*
Explicitly mentioning the CoI framework or its components	Lecturers Academic secretariat and IT Division	*"Thank you, Prof. A* *What are the good strategies to create a social presence with online learning like this? Of course, we also want students to feel "present" in class and be able to express, discuss, ask questions and so on. Do you think there are ways we can make our students more comfortable in class? What are your opinion, Professors?”* *[Lecturers]* *Good afternoon friends,* *Apart from communication between students and lecturers, we also provide communication facilities aimed at the secretariat and lecturers who can help convey information to students. We understand that communication is an important element of the OCL as a model of Community of Inquiry theory. Therefore, we always try to improve the quality of communication between elements of the faculty. Regards, IT Division* *[Academic secretariat and IT Division]*

Based on our thematic content analysis result, these are the characteristics of role-playing the students’ exhibit to answer RQ1:

- When participating in group discussions, students related to the current situation and their learning experiences based on the perspective of the roles they played.
- Very few students explicitly mentioned the CoI framework or its components. No students reminded their group of the learning objective: to improve their skills by implementing the CoI framework in online discussion. All groups placed greater focus on the challenges of implementing online collaborative learning.
- At the end of the discussion period, all groups produced sets of agreed ideas and concepts, but these were not compiled in the form of recommendation documents. However, the two groups summarised the results of their discussions. Only one group provided an in-depth rationale behind their recommendations and the arguments to support the recommendation (resolution).
- Students raised the subject of the COVID-19 pandemic concerning the conditions associated with the application of distance learning. Students conveyed experiences as online learners (particularly those experiences that were less enjoyable). The experiences were expressed according to their role. For instance, parents of students shared their child’s difficulties in learning in the current pandemic situation. Students presented their real experiences from the perspective of their role-play group.
- Students conveyed expectations of distance learning (from a student perspective). The expectations were expressed in the discussions as those of the role-play group. Moreover, they explained the situation that triggered the expectation. In the joint discussion and reflection session (following the role-playing activity), students were asked to share their experiences of implementing CoI. All students applied all of the CoI components. However, only a small number of students stated that they had consciously implemented the CoI framework explicitly.

### The pattern of the social, cognitive, and teaching presences

The pattern of presence of the CoI component between groups in 2019/2020 Even Semester class is the same. It is assumed that this pattern also applies in the other two classes. Therefore, the encoded and analysed transcripts only data from one semester, class of the second semester 2019/2020. A quantitative content analysis was carried out to answer the RQ2, SP, TP, and CP patterns in role-playing activities. The sample of discussion transcripts, consisting of 306 messages, was separately coded by two independent coders before comparison. We used Cohen’s kappa to measure the level of agreement between the coders (Cohen, 1960). The result is 0.9, and it indicates that the agreement between the coders was high. If disagreement occurred, the coding decision was discussed and agreed upon by both coders. The final decision is presented in Table 7.

**Table 7:** The frequencies of SP, TP, CP

The CoI components	Frequency
Social Presence (SP)	253
Teaching Presence (TP)	136
Cognitive Presence (CP)	225

**Fig. 1:**
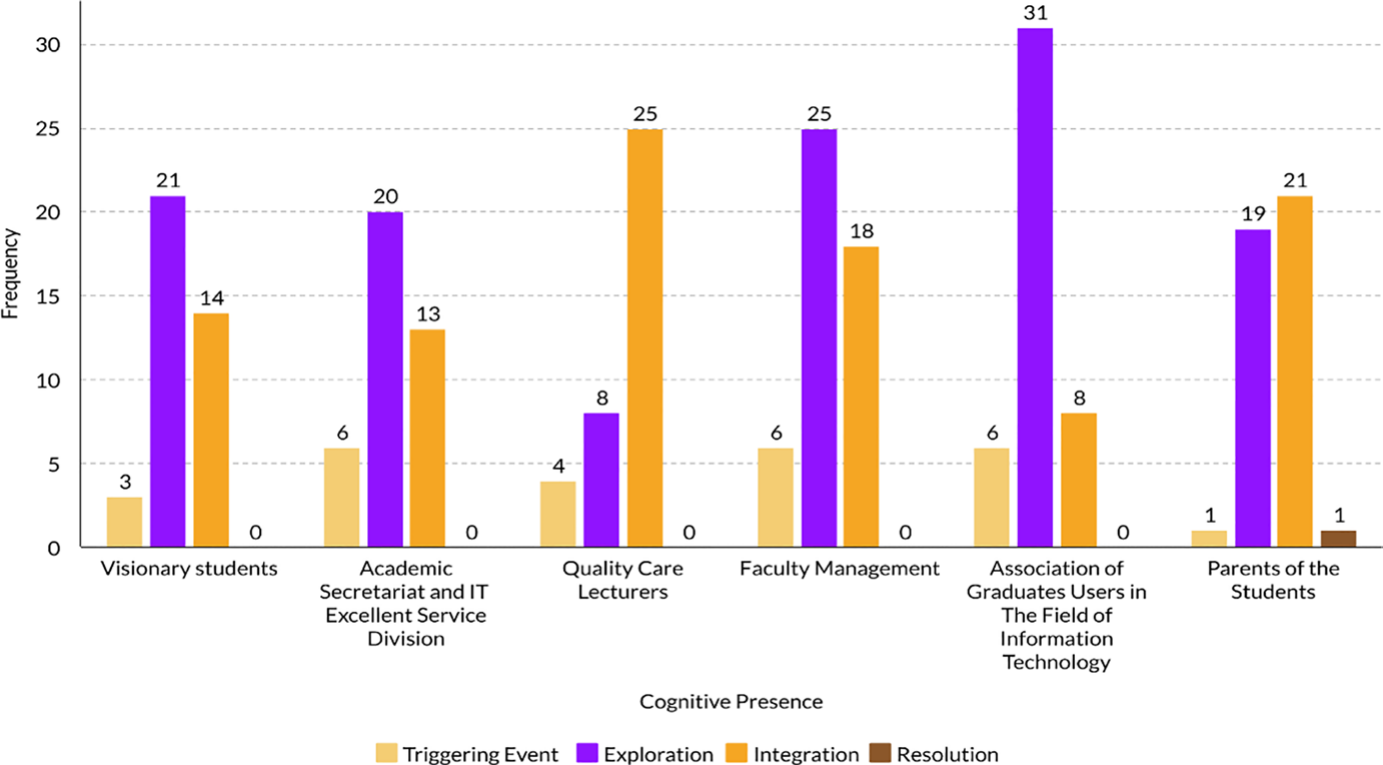
The frequencies of the sub indicators of CP (class of second semester 2019/2020)

SP had the highest frequency, demonstrating that students presented themselves socially in the form of greetings, name mentions and maintained group cohesion. CP describes how students solved problems collaboratively. The coding results, based on the sub-indicators of CP, are shown in Table 8 and Fig. 1.

**Table 8:** The frequencies of the sub indicators of CP

The CoI components	Categories	Frequency
Cognitive Presence (CP)	Triggering Event	26
	Exploration	124
	Integration	99
	Resolution	1

The most frequently exhibited sub-indicator was that of exploration (Fig. 1). Exploration appeared in the form of brainstorming and agreement without argument. Resolution proved the most difficult to attain. Meyer (2003) explained that integration and resolution are more demanding compared to exploration. To attain integration and resolution, students need to reflect on their experiences.

Based on our quantitative analysis, we found that the patterns of social presence, cognitive presence, and teaching presence for each group, answering RQ2, are:

- Four groups showed a similar pattern in the presentation of particular indicators of CP: they conducted fast triggering events, extremely intensive exploration, followed by less intensive integration. Two groups demonstrated a higher intensity of integration compared to exploration. The group of lecturers displayed a process of fast triggering events and exploration before proceeding to integration and synthesising of ideas.
- All groups tended to interpret problems rapidly and carried out considerable analysis and integration of ideas. Whether agreeing or disagreeing during discussions, students often provided arguments to support their thinking.
- The most challenging indicator to achieve is that of the resolution, as it requires the ability to apply solutions to different contexts or defend proposed solutions. As a generalisation, groups only proposed solutions in the form of recommendations and brief background information. Only one group achieved resolution; this group proposed a detailed background-supported solution, complemented by the impact of implementing the proposed recommendations and the consequences should they not be implemented.
- The explicit mention of CoI or its components is limited, occurring in less than five messages, and only two groups mentioned it: lecturers and academic secretariat, and IT Division. A lecturer discussed the use of CP in solving specific problems. An IT Division member argued the application of the CoI framework to improve the quality of communication in the faculty environment.
- Although it does not explicitly discuss the CoI framework, the application of SP, TP, and CP were performed by all groups; however, the essential sub-indicators of these presences have not all been applied. For example, students still lack TP in terms of reminding each other of the learning objectives, assessing the discussion progress, and summarising the discussion results. The dominant sub-indicator of TP is the directing of the discussion through asking questions.

### Students’ learning experience and satisfaction

The third research question explores the learning experience and student satisfaction. We analysed the data from the reflection of learning and lecturer evaluation questionnaires to answer the question.

#### Reflection

Students were asked to reflect on their learning experience, in a discussion board, after completing the role-playing session. 22 out of 63 students were voluntarily conveying their experiences. Based on the main ideas conveyed in the reflection, they can be grouped into three themes: obstacles faced, opinions about the application of the CoI framework, and opinions on the role-playing implementation.

#### Obstacles

Two students complained about the nested and complicated discussion board feature when the discussion went fast and long. Two students admitted that they were confused about how to play a role at the beginning because this was their first experience. One student was confused about motivating a group of friends to be active and direct the discussion without appearing dominant.

#### Students’ opinion

Thirteen students gave their opinions on the implementation of the CoI framework. All opinions are positive. Students feel the difference with their previous discussion experiences when they are not familiar with the CoI framework. It makes the discussion ‘livelier’ and more focused. Participants can avoid boredom by social presence.

Furthermore, it helps students map how they should appear in the forum. Hence, students realize what to do in the discussion and are more motivated to monitor their contribution to the discussion. Eight students gave positive comments on the learning process in general: it was fun to learn with a new perspective and a broad perspective to appreciate the learning process.

#### Lecturer evaluation by students

Lecturer Evaluation by Students is a questionnaire provided by the university to determine student perceptions of the implementation of learning at the end of the semester. The questionnaire consists of two parts. The first part asked about the students' perception of Learning Content, Teaching–Learning Process, Class Management, and Learning Evaluation with 6-Likert Scale items (1-Strongly Disagree, 2-Disagree, 3-Somewhat Disagree, 4-Somewhat Agree, 5-Agree, and 6-Strongly Agree).

All 63 students enrolled in the CAI class of 2^nd^ Semester 2019/2020 voluntarily answered the first part of the questionnaire. The overall average score in Part 1 is 5.65 shows that students are satisfied with the learning undertaken during the semester. Students scored well on every aspect: Learning Content, Teaching–Learning Process, Class Management, and Learning Evaluation with an average score of 5.7, 5.65, 5.59, and 5.66, respectively. Table 9 shows the results of the first part of the questionnaire about student perceptions in learning implementation.

**Table 9:** Lecturer evaluation by students: learning implementation

The components	Questions	Result	Average
Learning Content	Lecturers convey the teaching design (Lecture Unit / Syllabus / Teaching Design Book) clearly at the beginning of the lecture	5.66	5.70
	Learning materials are arranged systematically to make it easier to understand the interrelationships between the materials	5.75	
	Lecturers encourage students to actively participate in the learning process	5.68	
Teaching–Learning Process	Lecturers convey lecture material clearly	5.68	5.65
	Lecturers are able to create a classroom atmosphere that is conducive to learning	5.62	
	Lecturers are able to provide examples that help to understand difficult concepts	5.58	
	Lecturers are able to answer questions clearly	5.66	
	Evaluation of the learning given (for example: quizzes, Midterm Test, Final Test, assignments, etc.) in accordance with the teaching material	5.71	
	The weight of each component of the assessment is in accordance with the workload	5.66	
	The grades that I get represent my true abilities	5.68	
Class Management	Lectures are held on time	5.42	5.59
	Lecturers are open to receiving input from students	5.75	
	Lecturers apply teaching contract consistently	5.60	
Learning Evaluation	Evaluation of the learning given (for example: quizzes, Midterm Test, Final Examination, assignments, etc.) in accordance with the teaching material	5.69	5.66
	The weight of each component of the assessment is in accordance with the workload	5.66	
	The values ​​I get represent my true abilities	5.63	
Overall Average Score			5.65

The second part is an open-ended question to provide suggestions and criticism. The results can be seen in Table 10. In contrast to the Likert-Scale items, which all students answered, only 14 students wrote their opinions in the second part of the questionnaire. When the first part (quantitative) and the second part (qualitative) of questionnaires are compared, it can be concluded that most students' comments are good and even excellent. In addition, the quantitative components which are reflected by the qualitative data, are part of the Teaching–Learning Process and Class Management.

**Table 10:** Lecturer evaluation by students: suggestions, criticisms, ideas, and comments

Suggestions, criticisms, ideas, and comments
• One of the best courses and instructors
• Thank you, *Mr. A* and *Mrs. B*
• Thank you, *Mrs. A*., *Mr. B* for the learning experience
• Thank you
• Please note to the time the lecture ends so as not to exceed the time it should be
• I am very happy in learning this CAI course. I can learn a lot about what if in the future I want to build a website for computer-aided learning but also put forward existing learning theories. Here I have learned a lot from *Mr. A* and *Mrs.* about how to communicate well, improve critical thinking, etc. Hopefully *Mr. A* and *Mrs. B* are always healthy so they can still teach fun CAI classes again and maybe each meeting can be started with an interactive mini game to trigger students' pre-existing knowledge about what they have learned in the previous meeting:)
• Thank you very much for appreciating even out small contribution:))
• Thank you *Mr. A* and *Mrs. B*, cheer up, as always!
• *Mr. A* and *Mrs. B* are very good at teaching
• The best
• Students are required to be active in the CAI classes
• Very Good
• Thank you very much for the knowledge, *Mrs. B* and *Mr. A*. I am very grateful to be guided by extraordinary lecturers like both of you. Hopefully the knowledge gained in the class will give blessings for today, the following days and the hereafter. Thank you
• The learning methods applied to the CAI course are very representative of the learning theories we learn in this course, so that we experience the real implementation in our daily life. *Mr. A* and *Mrs. B* as lecturers always provide direction and feedback to us so that we know where the mistakes are and what should be improved. Thank you *Mr. A* and *Mrs. B*, I hope you will always be healthy

Note: The class was facilitated by two lecturers, represented in the text by their aliases: Mr. A and Mrs. B

If the students' reflections and their assessments on the Lecturer Evaluation by students, it can be concluded that both are consistent. Students had good learning experiences. In particular, reflection is related to the following components.

- Learning Content: Lecturers encourage students to participate in the learning process actively.
- Teaching–Learning Process: Lecturers can create a classroom atmosphere that is conducive to learning
- Class Management: Lecturers apply the teaching contract consistently.

In conclusion, we found some interesting findings related to students' perceptions of the role-playing learning experience and their learning satisfaction. Learning with role-playing is the first experience for students. Hence, there is a small number of students feel confused for a while at the beginning. Nevertheless, they can immediately adapt after seeing how other students carry out their roles. Moreover, students have positive experiences in role-playing, seeing problems from various sides, and getting hands-on experience. It showed in the quantitative and qualitative data of Lecturer Evaluation by Students. They feel benefited by studying the CoI framework and applying it in discussions. Students think the CoI framework directs how to manage good discussions. This experience makes students more aware of their role, thus monitoring the quality of their contribution.

## Discussion

The study revealed that that difference in the discussion duration (5 and 7 days) and the number of group members (9 or 10 students) did not correlate with the average number of students’ posting. However, the difference in student involvement in discussions might correlate with the academic load during the discussion or the difference in students’ preparedness.

Very few students explicitly mentioned the CoI framework or its components during the role-playing. No students reminded their group of the learning objective: to improve their skills by implementing the CoI framework in online discussion (teaching presence). In addition, teaching presence, which describes how students solved problems collaboratively, has the lowest frequency compared to social and cognitive presence. Students admitted that it was challenging to motivate and direct discussions. Students are not yet able to become online discussion facilitators. This is consistent with the previous studies on Linear Algebra classes (Junus et al., 2014; Junus et al., 2019). An intervention from an instructor is needed so that students apply TP or regulate the roles of group members; for example, there are leaders, facilitators, timekeepers.

In general, students had positive experiences with role-playing and thought that CoI guided them in the discussion. These acquired skills and knowledge need to be applied in online collaborative learning to other courses. These skills need to be nurtured in the following courses. In addition, students could manage online text dialogue and actively participate in reflective practice facilitated by online discussion forums and assignments.

Based on observations over the last three semesters, a set of similar challenges arise. These challenges affect the implementation of role-playing in asynchronous discussion forums. We believe that these challenges will lead to the failure of building the role-playing activities. For example, active discussion in role-playing activities usually takes time. In the beginning, some group members are unaware of the learning goals and advantages of activities. Therefore, they are oblivious to the fact that it has a significant effect on their learning. The lack of their engagement will affect badly in the role-playing processes. As a result, they will not achieve the learning objectives. These challenges were caused by the difficulty of familiarisation with the learning environment (Mclaughlan & Kirkpatrick, 2005).

Some strategies are recommended as anticipatory steps in designing role-playing activities. These strategies addressed teachers in implementing their role-playing activities. It emphasizes the role of teaching presence in learning discussion. These proposed strategies can be used as a guide to design role-playing activities. For example, teachers are advised to convey expectations and give provision of learning incentives explicitly. It will improve the students’ engagement in role-playing activities. Table 11 below explains these challenges and the proposed strategies.

**Table 11:** Challenges in the implementation phase and proposed strategies

The challenges	Proposed strategies to overcome the challenges
At the beginning of the discussion, group members are inactive Note: Possibly because they do not realise the learning objectives and the benefits of role-playing, or they do not see a direct impact on learning attainment	• Dissemination and modelling of social, teaching and cognitive presences • Remind students of the relevance and purpose of learning and the reasons for selecting the role-playing method • Explicitly convey expectations • Provision of learning incentives, such as demonstrating appreciation for student contributions and the value added to discussions, based on the process and results of the discussion
Asynchronous discussion is flexible. As a result, the learning process takes longer than scheduled for face-to-face. Role-playing requires a process of simulating the asynchronous online discussion and carrying out an assigned role	• Taking advantage of the flexibility of asynchronous discussion, such as giving more time to think deeply and prepare quality contributions
Students enjoy playing the assigned role; this results in the focus being drawn away from the learning goals, namely, applying the CoI framework Note: Students may be confused between the purpose of the role-playing activity and the expected output of the discussion: recommendations for faculty management in implementing OCL. It is possible that students do not fully understand the CoI framework, in particular, the sub-indicators and their important functions	• In the preparation stage of the role-playing, it is necessary to ensure that students understand the CoI framework, its sub-components and their important functions • Explain to students the purpose of the discussion and the expected outcome(s) • Remind students of the learning objective during the discussion • Explicitly remind students to apply every component and sub-indicator of social, teaching and cognitive presences, in accordance with the dynamics of the discussion
It is difficult to evaluate individual and group performance in online discussions, both in general and in role-playing. The provision of meaningful and timely feedback and maintaining learning motivation presents further challenges	• Provide opportunities for students to perform peer assessment (students assess the performance of their peers) • Provide rubric and its explanation
The success of individuals and groups depends on individual contributions and the ability of individuals to interpret and carry out their roles Groups whose discussions go well are those that have one or more active members. This dominant member indirectly inspires other members to respond. Groups that do not have a prominent member tend to be inactive	• Heterogeneous group formation • Encourage each group to select a leader, timekeeper and other possible roles needed • Encourage the sharing responsibility

## Conclusion

This current study investigates the use of online role-playing in learning the CoI framework. The objective is for students to be able to apply the CoI framework in an asynchronous online collaborative learning environment. The participants implemented all the components of the CoI framework, but improvements are needed in evaluating and directing the discussions. The groups’ patterns of SP, TP, and CP are similar; however, SP proved the easiest to perform, and students could feel the effects immediately. The levels of critical thinking shown by students did not reach the level of resolution.

Students performed their roles and focussed on conveying ideas based on the perspective of the role assigned to them. The focus group produced the output of the discussion but did not focus on the application of all the CoI framework sub-indicators. In theory, it is easier to play one’s own role than it is to act out the role of others. Despite this, however, the results indicated that those playing the role of student were neither the most active nor did they possess the longest average message length. Those roles with more issues to discuss tended to have longer message lengths. Observations discovered that the presence of a dominant member influences group activity – dominant members stimulate others to be active.

Based on the findings, increased intervention from the class lecturer is recommended, explicitly in directing groups to intentionally apply the CoI framework and raise awareness of the learning objectives. It is also recommended that students are given more preparation time before role-playing activities commence; in particular, students should be given more time to learn the CoI framework and provided with enough examples in its application.

This study has limitations that paved the way for future research topics. First, the instructors provided no treatment intervention during the role-playing. The study indicated that instructor intervention is required to help students apply TP. Second, another significant aspect for further consideration is group formation in online role-playing that utilises small group discussion forums. Future research topics include role-playing with increased intervention, strategies to improve the students’ preparedness to collaborate online, and how role-playing improves critical thinking, learner engagement.

## Data Availability

The data presented in this study are available on request from the corresponding author. The data are not publicly available due to the confidential information.
